# Using giant scarlet runner bean embryos to uncover regulatory networks controlling suspensor gene activity

**DOI:** 10.3389/fpls.2015.00044

**Published:** 2015-02-06

**Authors:** Kelli F. Henry, Robert B. Goldberg

**Affiliations:** Department of Molecular, Cell and Developmental Biology, University of California, Los Angeles, Los Angeles, CA, USA

**Keywords:** *Phaseolus coccineus*, scarlet runner bean, suspensor, gene regulatory network, *cis*-regulatory elements, transcriptome, comparative genomics

## Abstract

One of the major unsolved issues in plant development is understanding the regulatory networks that control the differential gene activity that is required for the specification and development of the two major embryonic regions, the embryo proper and suspensor. Historically, the giant embryo of scarlet runner bean (SRB), *Phaseolus coccineus*, has been used as a model system to investigate the physiological events that occur early in embryogenesis—focusing on the question of what role the suspensor region plays. A major feature distinguishing SRB embryos from those of other plants is a highly enlarged suspensor containing at least 200 cells that synthesize growth regulators required for subsequent embryonic development. Recent studies have exploited the giant size of the SRB embryo to micro-dissect the embryo proper and suspensor regions in order to use genomics-based approaches to identify regulatory genes that may be involved in controlling suspensor and embryo proper differentiation, as well as the cellular processes that may be unique to each embryonic region. Here we review the current genomics resources that make SRB embryos a compelling model system for studying the early events required to program embryo development.

## WHY STUDY THE SUSPENSOR?

Embryogenesis in most higher plants begins with a double fertilization event, in which one sperm cell fertilizes the egg cell to form the zygote, and the other fertilizes the central cell to form the endosperm (Bleckmann et al., 2014). The zygote undergoes an asymmetric cell division, giving rise to a small, cytoplasm-rich apical cell and a large, vacuolated basal cell (West and Harada, 1993). The apical cell divides to form the embryo proper, which becomes the next generation plant, whereas the basal cell divides to form the suspensor, a terminally differentiated structure that transports nutrients to the embryo proper (Yeung, 1980; Nagl, 1990) and degenerates as the embryo matures (Yeung and Meinke, 1993). The uppermost cell of the suspensor, the hypophysis, contributes to the root meristem of the embryo (Dolan et al., 1993). While much is known about embryo proper development, comparatively little is known about the suspensor (Lau et al., 2012; Wendrich and Weijers, 2013). Genetic studies in *Arabidopsis* have illuminated some processes leading to suspensor differentiation. The molecular pathways involved in elongation of the zygote, the asymmetric division that forms the two-cell embryo, and apical and basal cell fate specification require (1) auxin signaling (Friml et al., 2003), (2) the YDA/MAPK signaling pathway (Bayer et al., 2009), and (3) the transcriptional networks involving RKD4 (Waki et al., 2011), WRKY2, WOX2, WOX8, and WOX9 (Ueda et al., 2011). However, genes in these pathways account for a very small percentage of the ∼11,000 diverse mRNAs detected in the *Arabidopsis* suspensor (Belmonte et al., 2013), and the molecular mechanisms governing suspensor development and function remain largely elusive. In addition, little is known about (1) the regulatory networks controlling suspensor differentiation and development in species with diverse suspensor morphologies, (2) the mechanisms activating different gene sets in the embryo proper and suspensor after fertilization, and (3) the cellular processes governing suspensor degeneration in later embryo development.

## WHY USE SRB TO STUDY SUSPENSOR DIFFERENTIATION?

**FIGURE 1:**
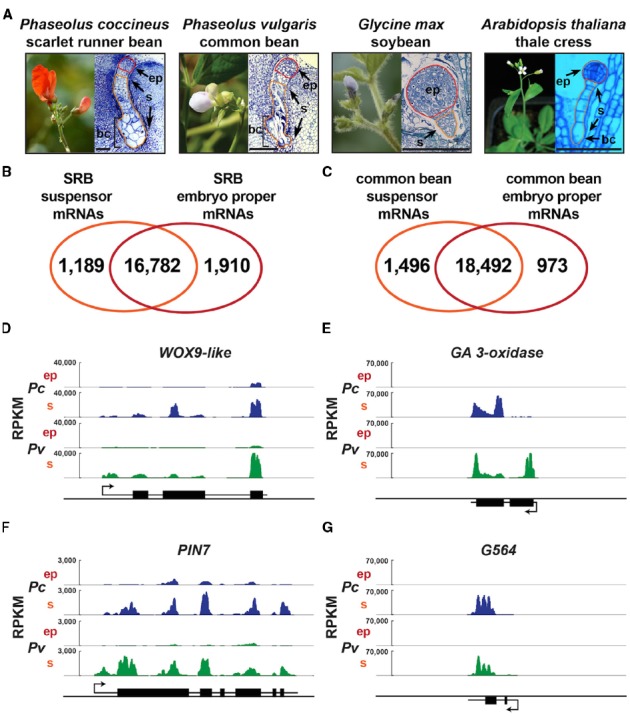
**Suspensors with diverse morphologies and bean suspensor-specific gene activity. (A)** Scarlet runner bean, common bean, soybean, and *Arabidopsis* plants, plastic sections of globular-stage embryo of SRB, common bean and *Arabidopsis*, and paraffin section of globular-stage embryo of soybean. Common bean flower image was taken from http://www.pbase.com/valterj/dsch9 photographed by Valter Jacinto, and *Arabidopsis* flower image was taken from Kawashima and Goldberg (2010). **(B,C)** Venn diagrams representing the mRNAs detected in SRB **(B)** and common bean **(C)** suspensor and embryo proper. RNA-Seq data for SRB and common bean are from GEO accession GSE57537. **(D–G)** Genome browser views of WOX9-like, GA 3-oxidase, PIN7 and G564 mRNA accumulation levels in SRB and common bean suspensor and embryo proper. Each panel depicts a 5 kb window including the gene structure. Black boxes represent exons. Black lines represent UTRs and introns. Arrows indicate the transcription start site. bc, basal cell(s); ep, embryo proper; RPKM, reads per kilobase per million; Pc, *P. coccineus*; Pv, *P. vulgaris*; s, suspensor. Scale bar: 100 μm.

The physical features of the SRB suspensor (Figure 1A), including its massive size, enlarged basal cells, and polytene chromosomes (Nagl, 1962) provide a unique system to study the functional significance of this highly specialized suspensor, the cellular processes shared by all suspensors, and suspensor differentiation events. Additionally, SRB seeds are a protein-rich legume crop, closely related to soybean, common bean, and cowpea in the economically important *Phaseoleae* clade of legumes, and thus can serve as a model for legume seed development. Common bean (*Phaseolus vulgaris*), which is a major source of calories in many developing countries^1^ and a $1B crop in the United States^2^, and SRB are congeneric species that diverged less than eight million years ago (mya; Lavin et al., 2005) and can form successful hybrids (Lamprecht, 1941; Thomas, 1964), as was first reported by Mendel in 1865 (cited by Mok et al., 1986). SRB diverged ∼19 mya from soybean (Lavin et al., 2005), the second largest crop in the United States (see text footnote 2). Taken together, SRB is an excellent plant in which to study suspensor development because of (1) its specialized structure, (2) its phylogenetic placement in the legume family, (3) a 40-year history of use as a model for embryo development (Yeung and Meinke, 1993; Kawashima and Goldberg, 2010), and (4) new genomic resources, including (i) the common bean genome sequence (Schmutz et al., 2014) and (ii) gene expression profiles for the SRB suspensor and embryo proper during early embryogenesis (Le et al., 2007; Kawashima and Goldberg, 2010; GEO accession GSE57536).

## WHAT WAS LEARNED FROM USING SRB AS A MODEL FOR SUSPENSOR DEVELOPMENT FOR OVER 40 YEARS?

The first experimental studies of suspensor function were performed by Ian Sussex and collaborators using SRB because the large size of the SRB embryo allows hand-dissection of the suspensor and embryo proper, and facilitates the collection of large amounts of suspensor tissue for use in biochemical studies (Clutter and Sussex, 1968; Walbot et al., 1972; Sussex et al., 1973; Clutter et al., 1974; Lorenzi et al., 1978). Early SRB experiments determined that the suspensor is required for the development of the embryo proper (Cionini et al., 1976; Yeung and Sussex, 1979), and that it is highly transcriptionally and translationally active (Walbot et al., 1972; Sussex et al., 1973; Clutter et al., 1974), in part due to its polytene chromosomes which can increase the DNA content of suspensor cells up to 8,192C (Nagl, 1962; Brady, 1973). There is a progressive increase in the level of polyteny from the chalazal pole of the suspensor to the large basal cells (Figure 1A; Brady, 1973). Although the biological function of polytene chromosomes and their puffs and loops in SRB suspensor cells (Nagl, 1970, 1974) is unclear, polyteny is a sign of terminally differentiated, highly specialized tissues such as *Drosophila* salivary glands (Heitz and Bauer, 1933).

Two specialized suspensor functions uncovered from early SRB studies are transport and hormone biosynthesis. The giant basal cells of the suspensor function as “transfer cells,” using their enlarged membrane surfaces and prominent ingrowths to absorb solutes from the surrounding seed tissues and transport them to the growing embryo proper (Gunning and Pate, 1969; Nagl, 1974, 1990; Yeung and Sussex, 1979; Yeung, 1980). The SRB suspensor not only acts as a conduit for nutrients, but also synthesizes growth regulators, e.g., gibberellic acid (GA) needed by the embryo proper in early development (Alpi et al., 1975). In fact, biochemical studies showed that SRB suspensors are a rich source of GAs (Alpi et al., 1975) and contain enzymes for synthesizing GAs (Ceccarelli et al., 1979, 1981). Classical approaches carried out 40 years ago revealed that the transport of nutrients and GA biosynthesis are essential processes carried out by the SRB suspensor for embryo development.

## HOW HAS GENOMICS BEEN USED TO DISSECT EARLY SRB SUSPENSOR DIFFERENTIATION AND DEVELOPMENT?

Because the SRB embryo is uniquely large, our laboratory was able to hand dissect globular-stage embryo-proper and suspensor regions and use pre-NextGen sequencing approaches—such as differential display, in situ hybridization, EST sequencing, and microarray analysis—to study the gene expression events that occur shortly after fertilization (Weterings et al., 2001; Le et al., 2007; Kawashima and Goldberg, 2010; Le, 2013). These experiments showed that the SRB embryo apical and basal regions transcribe different genes as early as the four-cell stage, suggesting that these regions are specified at the molecular level after division of the zygote (Weterings et al., 2001). At the globular stage there is a large overlap in genes expressed in the embryo proper and suspensor regions that are derived from the apical and basal cells, respectively (Le et al., 2007). Many suspensor-specific SRB genes were identified, however, including (1) all genes in the GA biosynthesis pathway, (2) a *WOX9-like* homeodomain transcription factor gene (*PcWox9-like*), and (3) *PcG564*, a gene of unknown function, among many others (Weterings et al., 2001; Le et al., 2007; Kawashima and Goldberg, 2010; Le, 2013; Henry, 2014). We confirmed these observations by using laser-capture micro-dissection (LCM) technology to collect SRB globular-stage embryo proper and suspensor regions with more precision (Le et al., 2007), RNA-Seq for transcriptome profiling (GEO accession GSE57536), and the common bean (*Phaseolus vulgaris*) as a reference genome (Schmutz et al., 2014; Figure 1). The genome browser view illustrates the up-regulation of *PcGA 3-oxidase*, *PcG564*, and *PcWox9-like* genes in the SRB suspensor, in addition to the *PcPIN7* auxin transporter gene that has been shown by others to be up-regulated in the *Arabidopsis* suspensor and play an essential role in establishing apical-basal polarity (Friml et al., 2003; Figures 1D–G).

Knowing the spectrum of transcription factor genes that are active in the embryo proper and suspensor is a first step to building gene regulatory networks that program embryo development. One or more mRNAs unique to each embryo region could encode transcription factors that are directly linked to the processes by which these two regions of the embryo activate different gene sets shortly after fertilization and become specified for different developmental fates (Weterings et al., 2001). Our strategy of working backward from globular-stage gene activity to cell-fate specification is particularly amenable to the suspensor because its differentiation precedes that of the embryo proper, and the suspensor cells are direct clonal descendants of the basal cell of the two-cell embryo (Weterings et al., 2001; Kawashima and Goldberg, 2010). Thus, the factors that activate genes in the suspensor might be directly linked to the basal cell specification mechanism. For example the globular-stage expression pattern of the SRB *PcWOX9-like* gene is remarkably similar to its *Arabidopsis* counterparts *AtWOX8* and *AtWOX9* (Haecker et al., 2004). In *Arabidopsis*, WOX8 mRNA accumulates in the zygote, and is then confined to the basal cell of the two-cell embryo and the globular-stage suspensor (Haecker et al., 2004). *AtWOX8* transcription is regulated, in part, by the WRKY2 transcription factor (Ueda et al., 2011). Thus, the WRKY2-WOX8 pathway functions in establishing zygote polarity by initiating a shift in organelle positions in the zygote enabling asymmetric division to occur (Ueda et al., 2011). Identifying the downstream target genes of *PcWOX9-like*, and other SRB suspensor-specific transcription factors, should facilitate building regulatory networks that program suspensor gene activity and uncovering the cellular events that are responsible for suspensor differentiation (Le et al., 2007).

## WHAT HAS BEEN LEARNED FROM USING COMPARATIVE GENOMICS TO IDENTIFY CONSERVED SUSPENSOR FUNCTIONS?

The suspensor is an evolutionarily conserved structure present in most seed-bearing plants and even some mosses, which diverged ∼425 mya (Wardlaw, 1955; Kawashima and Goldberg, 2010). To understand more broadly the core functions carried out by all suspensors, the transcriptomes of suspensors from various species can be compared to identify conserved metabolic processes and transcription factors that may regulate conserved suspensor functions. We have previously reported that *Pc*G564 mRNA is also localized specifically in the basal region and suspensor of a transgenic globular-stage tobacco embryo transformed with an intact *PcG564* gene (Weterings et al., 2001). This shows that the suspensor transcriptional machinery regulating *PcG564* expression is conserved in plants that diverged ∼150 mya (Paterson et al., 2004). It remains to be determined what other transcription factors are conserved in the suspensors of divergent species and what their downstream target genes are.

We have laid the foundation for a comparative genomics analysis of the SRB suspensor transcriptome with that of common bean, soybean, and *Arabidopsis*. Our laboratory has used LCM and RNA-Seq to profile the globular-stage suspensor and embryo proper transcriptomes of SRB and common bean (GEO accession GSE57537; Figure 1). WOX9-like, GA3-oxidase, PIN7 and G564 mRNAs are up-regulated similarly in both SRB and common bean suspensors (Figures 1D–G), demonstrating the conservation of gene activity and cellular functions carried out by giant bean suspensors. In collaboration with John Harada’s laboratory at UC Davis, we have profiled the transcriptomes of the suspensor and embryo proper of soybean (*Glycine max*; GEO accession GSE57349) and *Arabidopsis* (Belmonte et al., 2013) embryos. Recently Slane et al. (2014) profiled *Arabidopsis* globular-stage embryo proper and suspensor nuclear transcriptomes using fluorescence-activated nuclear sorting (FANS). These datasets should illuminate several important questions regarding higher plant suspensors. What functions are conserved in the SRB and common bean giant suspensors? What are the functions of conserved transcription factors in legume suspensors? What functions are evolutionarily conserved in all suspensors regardless of size, morphology, or specialized function?

## WHAT UNIQUE PROCESSES OCCUR IN GIANT BEAN SUSPENSORS THAT DIFFER FROM LESS SPECIALIZED SUSPENSORS?

Suspensors display a wide range of morphological diversity in higher plants (Kawashima and Goldberg, 2010; Figure 1A). For example, closely related legume species, soybean and SRB, have distinct suspensors. The soybean suspensor is small, consisting of a few cells, whereas the SRB suspensor is huge containing ∼200 cells (Sussex et al., 1973). The *Arabidopsis* suspensor, which is even smaller than that of soybean, is a single file of 7–10 cells. There may be several biological processes unique to giant bean suspensors and absent in smaller suspensors, such as those of *Arabidopsis* and soybean. One of the first characterized functions of the SRB suspensor, the synthesis of GA, may be unique to giant, highly specialized bean suspensors (Kawashima and Goldberg, 2010). In fact, GA also accumulates in the massive suspensor of the legume *Cytisus laburnum* (Picciarelli et al., 1984). While *GA 3-oxidase* mRNA (encoding the last enzyme in the GA biosynthesis pathway) accumulates to a high level in both SRB and common bean suspensors at the globular stage (Figure 1E; Solfanelli et al., 2005), mRNAs representing the *Arabidopsis* homologs of *GA 3-oxidase* do not accumulate in the suspensor; instead, they accumulate in the endosperm of globular-stage seeds (Belmonte et al., 2013). In dicots, the suspensor and the endosperm are both short-lived structures that degenerate once they have accomplished their function of nourishing the developing embryo proper. It has been suggested that in species with massive suspensors, such as SRB and common bean, the suspensor takes over endosperm functions, resulting in delayed endosperm cellularization and a decreased amount of endosperm (Tison, 1919; Schnarf, 1929; Newman, 1934; Lorenzi et al., 1978; Guignard, 1880). Although there are specific examples that do not support this hypothesis in all plants (Lersten, 1983), it may apply in some cases. Thus, the endosperm GA biosynthesis gene regulatory network in *Arabidopsis* might have been co-opted by the giant bean suspensors, or vice versa. In *Arabidopsis* seeds, only the location of GA hormone synthesis has changed relative to giant bean seeds, not the developmental time at which hormone accumulation occurs. Perhaps the site of GA synthesis within the seed is not important, as long as the hormone is transported to the embryo proper at the globular stage of development.

Comparative studies of the gene regulatory networks controlling the development and differentiation of suspensors of divergent species will help to unlock the changes that occurred in evolution to produce morphologically and functionally distinct suspensors. A change in gene expression between species could be attributed to an alteration in a transcription factor protein, but more commonly it has been shown to result from changes in gene promoters (Pina et al., 2014). Identifying functional *cis*-regulatory elements and transcription factors that program suspensor gene activity, and comparison between different species will help to trace how novelties arose in gene regulatory networks, which may have led to the evolution of morphologically and functionally distinct suspensors across species.

## WHAT ARE THE *CIS*-REGULATORY ELEMENTS CONTAINED WITHIN THE GENOME THAT PROGRAM SUSPENSOR-SPECIFIC TRANSCRIPTION?

**FIGURE 2:**
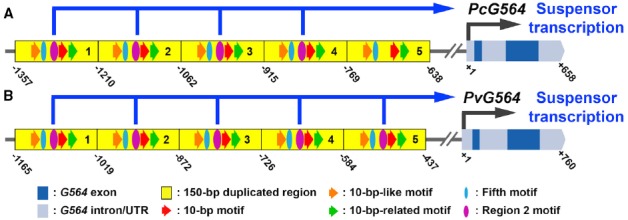
**Regulatory elements controlling suspensor-specific gene expression. (A,B)** Conceptual representations of the *G564* gene and upstream region in SRB **(A)** and common bean **(B)** taken from Henry (2014). Suspensor *cis*-regulatory sequences were identified by functional *PcG564* promoter/*GUS* gene fusion and mutagenesis experiments in transgenic tobacco (Kawashima et al., 2009; Henry, 2014). Dark blue boxes represent exons. Light blue boxes represent UTRs and introns. Yellow boxes represent 150-bp tandem repeats in the upstream region (Kawashima et al., 2009). Red, orange and green arrows indicate the 10-bp motif, 10-bp-like motif and 10-bp-related motif. Purple ovals indicate the Region two motif. Blue ovals indicate the Fifth motif. Numbers indicate positions relative to the transcription start site (+1). Pc, *P. coccineus*; Pv, *P. vulgaris*.

DNA sequence comparisons between related species have the potential to identify *cis*-regulatory elements that may regulate suspensor-specific gene transcription (Haeussler and Joly, 2011). However, wet-bench studies are required to determine whether predicted suspensor *cis*-regulatory elements are functional. Previously, we identified five *cis-*regulatory elements in the upstream region of the *PcG564* gene (Figure 2A) that activate transcription in transgenic tobacco and *Arabidopsis* suspensors (Kawashima et al., 2009; Henry, 2014). It remains unknown what other genes are regulated by *PcG564* suspensor *cis*-regulatory elements. The simplest hypothesis is that SRB suspensor up-regulated genes, such as *PcGA 20-oxidase* and *PcWOX9-like* (Le et al., 2007), are activated by the same suspensor *cis*-regulatory elements. Indeed, the *PcG564* suspensor *cis*-regulatory elements are found in the *PcGA 20-oxidase* and *PcWOX9-like* gene upstream regions (Kawashima et al., 2009; Henry, 2014), suggesting that these genes may comprise a suspensor gene regulatory network.

The common bean genome sequence (Schmutz et al., 2014) allows us to scan the upstream regions of all suspensor-specific genes for the presence of the five known suspensor *cis*-regulatory elements identified in *PcG564*. The common bean genome sequence can be used as a surrogate for the SRB genome because the two species diverged relatively recently (Lavin et al., 2005) and have similar gene expression profiles for the suspensor and embryo proper at the globular stage (Figures 1B–G). For example, G564 mRNA is up-regulated in the suspensor of both SRB and common bean relative to the embryo proper (Figure 1G), and the *G564* upstream region is highly conserved in these two species (Figure 2; Henry, 2014). The *PcG564* and *PvG564* upstream regions contain five tandem repeats of 150-bp, and each repeat contains the five known suspensor *cis*-regulatory elements, with the exception of the fifth repeat in *PcG564* (Figure 2). The suspensor *cis*-regulatory elements most likely function in *PvG564* because motif sequences and *G564* expression patterns are conserved in both bean species. The identities of the *trans*-acting factors that bind to the bean *G564* suspensor *cis*-regulatory elements remain a mystery. What other genes are regulated by the transcription factors that activate *G564*, what additional regulatory circuits control suspensor gene activity, and how these regulatory circuits are activated after fertilization remain unanswered questions.

## FUTURE PERSPECTIVES

The sequence of the common bean genome opens the door to *Phaseolus* suspensor gene regulatory network analysis on a genome-wide scale. Comparison of SRB and common bean suspensor transcriptomes with their embryo proper counterparts can identify suspensor-specific mRNAs that may be involved in processes specific to suspensor differentiation (Figure 1). The next major step is to identify suspensor-specific transcription factors, and determine their binding sites across the genome using, for example, ChIP-Seq. The power of the SRB system lies in its giant suspensor and polytene chromosomes, which can facilitate chromatin collection. Functional analysis of binding sites will also have to be carried out through promoter studies, as was done for *PcG564*, because transcription factor occupancy does not necessarily predict enhancer function *in vivo* (Peter and Davidson, 2009; Sanalkumar et al., 2014). The giant bean suspensor system has been resurrected, and should reveal new clues regarding processes that control suspensor differentiation and function in the near future.

### Conflict of Interest Statement

The authors declare that the research was conducted in the absence of any commercial or financial relationships that could be construed as a potential conflict of interest.

## Footnotes

1 http://faostat.fao.org

2 http://www.nass.usda.gov
